# A Resource Service Model in the Industrial IoT System Based on Transparent Computing

**DOI:** 10.3390/s18040981

**Published:** 2018-03-26

**Authors:** Weimin Li, Bin Wang, Jinfang Sheng, Ke Dong, Zitong Li, Yixiang Hu

**Affiliations:** 1 School of Information Science and Engineering, Central South University, Changsha 410083, China; weiminli@csu.edu.cn (W.L.); wb_csut@csu.edu.cn (B.W.); dongke@csu.edu.cn (K.D.); huyixiang@csu.edu.cn (Y.H.); 2 School of Information, Hunan University of Humanities, Science and Technology, Loudi 417000, China; 3 Department of Electrical and Electronic Engineering, The University of Hong Kong, Hong Kong SAR, China; u3544658@connect.hku.hk

**Keywords:** Internet of Things, transparent computing, industrial IoT, IoT OS

## Abstract

The Internet of Things (IoT) has received a lot of attention, especially in industrial scenarios. One of the typical applications is the intelligent mine, which actually constructs the Six-Hedge underground systems with IoT platforms. Based on a case study of the Six Systems in the underground metal mine, this paper summarizes the main challenges of industrial IoT from the aspects of heterogeneity in devices and resources, security, reliability, deployment and maintenance costs. Then, a novel resource service model for the industrial IoT applications based on Transparent Computing (TC) is presented, which supports centralized management of all resources including operating system (OS), programs and data on the server-side for the IoT devices, thus offering an effective, reliable, secure and cross-OS IoT service and reducing the costs of IoT system deployment and maintenance. The model has five layers: sensing layer, aggregation layer, network layer, service and storage layer and interface and management layer. We also present a detailed analysis on the system architecture and key technologies of the model. Finally, the efficiency of the model is shown by an experiment prototype system.

## 1. Introduction

As an emerging field of technology, Internet of Things (IoT) has aroused the great concern of governments, research institutions and enterprises. The term IoT was coined in 1999 by Kevin Ashton, who aims to connect different things over the networks. Currently, the ‘Things’ can be Radio Frequency IDentification (RFID) tags, sensors, actuators, mobile phones, and lightweight wearables, even virtual entities that can be uniquely identified [1]. Although the definition of ‘Things’ has changed as technology evolved, the essential attribute that they are enabled to interact with each other and cooperate with their neighbors to reach common goals without the aid of human intervention remains the same. The expected huge number of interconnected things, the interaction between things and high-performance computing, storage center and increasingly intelligent IoT devices open up new opportunities to create a smarter environment [2]. The industrial IoT uses IoT technologies in the industrial systems to gather real-time data, control the manufacturing environment and monitor the environmental metrics such as noxious gas, temperature, humidity and fire alarms. It can greatly improve the efficiency of manufacturing and reduce the cost of the enterprise. Hence, there is a growing interest in using IoT technologies in various industries. A number of industrial IoT projects have been conducted in areas such as agriculture, manufacturing and processing industry, environmental monitoring, and mine safety monitoring [3,4]. Industrial IoT devices range from tiny environmental sensors to complex industrial robots, and they are primarily sensors, controllers and special equipment that can accommodate harsh and complex industrial fields. IoT applications focus on collecting and processing the sensing and decision data in industrial environments and carrying out a large number of notifications [5].

However, the limitations of associated devices in the IoT in terms of storage, network and computing, and the requirements of large-scale data analysis and processing require a technology like Cloud Computing to supplement this field. Cloud Computing promises high reliability and scalability to provide ubiquitous access, dynamic resource scheduling and composability required for the IoT applications [6]. The authors of [7] present a detailed discussion on a novel CloudIoT paradigm where Cloud and IoT are merged together, involving completely new applications, challenges, and research issues. Due to the benefits from Cloud Computing, IoT’s service capabilities have been greatly expanded, and there are several projects and strong research efforts in this field. It seems that their integration is foreseen as an excellent IoT solution focused on by researchers [8].

Transparent Computing (TC) [9], which can be viewed as a special kind of Cloud Computing, provides a feasible solution to solve the above problems. TC is a new user-centric service mode whose aim is to provide users with unconscious and transparent services. However, unlike Cloud Computing, in TC, not only the applications (APPs) and user data, but also the underlying operating systems (OSs) are stored and managed at the server side, while the client does not store any OSs and APPs locally. They will be dynamically scheduled from the server to the client for execution in a block-streaming way according to users’ requests. In such a service mode, users simply just accomplish their tasks on the device with less CPU power and memory and do not consider the heterogeneous software details, thus providing across-platform and across-OS service for different kinds of terminals. On the other hand, TC can reduce the burden of the server and improve the system maintainability and security [10]. Thus, TC has attracted significant attention from both academia and industry and its applications are increasingly widespread over the past decade.

IoT being characterized by a very high heterogeneity of devices, protocols and Cloud platforms as well as widespread application of IoT OS [11] in the near future, it provides great opportunities to exploit TC to build more efficient, scalable and secure IoT service mode. First of all, TC can enable services to be executed on heterogeneous IoT devices from the remote server by decoupling the software stack from various devices, thus eliminating the dependency of the hardware and software on the industrial IoT device. Moreover, heterogeneous resources (including OSs, hlAPPs and private data) of various industrial IoT devices can also be stored and managed in a centralized way with TC. Therefore, TC improves the manageability, scalability and reliability of various types of industrial IoT devices and their resources. Finally, with the growing popularity of IoT, more and more information processing and decision-making tasks need to be executed on the IoT terminal device. Thus, they require greater intelligence processing capacity, which provides a favorable condition for the local execution scheme of transparent computing. On the other hand, the industrial IoT application environments also bring new challenges for applying TC into them, e.g., how to design an efficient and scalable solution to implement TC on the heterogeneous (including hardware and software) and resource-constrained IoT devices.

Our research group is conducting some important research issues about the integration of TC and industrial IoT. This paper proposes a novel paradigm for the industrial IoT where TC are integrated by means of a case study about Six-Hedge underground systems (referred to as Six Systems) [12]. The Six Systems is actually a comprehensive IoT platform in the underground mine, which includes: (1) a personnel regional positioning system; (2) a monitoring and supervision system; (3) an emergency refuge system; (4) a compressed air self-help system; (5) a water rescue system; and (6) a communication system. All six of the systems mentioned above are based on pervasive sensing, sensor data collection, data analysis and processing, and prevent accidents by locating the position of the underground staff and detecting hazardous gases. Therefore, the construction of Six Systems in underground mines is enforced by the government [13]. Based on the case study, we try to extend the idea of transparent computing to the industrial IoT and provide a novel and feasible solution for its resource services. The service model aims to provide unified and centralized storage, management and remote access service for the various heterogeneous resources of industrial IoT devices, especially operating system resources. We give a detailed description and analysis on the architecture of the model, and implement a prototype system for the new paradigm, and finally evaluate the performance. The experimental results show that the proposed model can provide efficient, scalable and reliable resource services for a wide variety of industrial IoT devices, while maintaining the comparatively ideal performance.

The rest of the paper is organized as follows. Section 2 explains the related work on some network computing applications in the IoT and transparent computing. In Section 3, the architecture of the resource service model for future industrial IoT based on TC and its characteristics are introduced. Section 4 presents key technologies of the model. Section 5 gives the experimental performance analysis of the specific key technologies in the model, and explains qualitative analysis of the effect on the future industry IoT. Finally, Section 6 includes conclusions and our future work.

## 2. Related Works

Internet of Things, which explains how many objects that surround us can be associated with the Internet and provide intelligent services for end users, has provided a promising opportunity to build powerful industrial systems and applications. With the advances in high-speed wireless communication, smart phones, wearable devices and network technologies, more and more networked things or smart objects are involved in IoT [2,3]. In order to provide high-quality services to end users and dominate the market, IoT’s technology depends on two key aspects in terms of standardization and the data storage and processing. On the one hand, the lack of standards is actually considered a big issue by most IoT researchers. Standardization of protocols, architectures and Application Programming Interfaces (APIs), which provides interconnection, interoperability and compatibility among heterogeneous smart objects, play a pivotal role in solving this problem [14,15]. On the other hand, IoT systems can generate massive heterogeneous data rapidly so that the data must be stored efficiently and processed with a high throughput [16]. Cloud Computing can provide an environment to enable resources to share in terms of infrastructure, data storage and analysis and software development platforms. Thus, Cloud based storage and processing solutions are becoming increasingly popular in the IoT systems. The integration of Cloud Computing and IoT solves such problems such as IoT’s limitations, data access, computing and data analysis, and can create new paradigms like Thing as a Service [17] and Sensor as a Service [18], which enable the Cloud to ubiquitously manage the remote sensors and smart things. The key issues and their respective potential solutions about IoT and Cloud Computing integration, known as Cloud of Things, were highlighted in [8]. The integration of agents and Cloud computing was proposed in [19], in order to implement a smart objects-oriented IoT architecture. The authors of Díaz et al. [6,7] provided a literature survey on the integration of Cloud and IoT, which involves completely new applications, challenges and research issues.

There are also some other computing paradigms that were integrated with IoT. A new platform, called Fog Computing, extends the Cloud Computing to the edge of the network and brings it closer to IoT devices, thus supporting IoT applications characterized by latency constraints, mobility and geo-distribution [20]. Context-aware computing, which can help IoT middleware to better understand sensor data and decide what data needs to be processed, has been identified as an important IoT research area by the European Union [21]. However, the integration of Cloud Computing and IoT is used most widely. Meanwhile, we note that IoT OS and the Cloud platform are bounded together. In other words, the devices run an IoT OS can only access the specified Cloud platform. Such a sealing property caused that IoT users can not access cross-OS and cross-platform services.

A client/server or SOA (Service-Oriented Architecture) architecture with ESB (Enterprise Service Bus) is powerful and efficient enough for massive sensing applications [22,23]. However, we also note that various IoT operating systems have been emerging over the last two years, including ARM (Advanced RISC Machines)’s mbed [24], a friendly Operating System for IoT-RIOT [25], Microsoft’s Windows 10 (Redmond, WA, USA) IoT [26], Google’s Brillo (Mountain View, CA, USA) [27] and Linux Foundation’s (San Francisco, CA, USA) Zephyr [28], etc. These IoT OS aim to run on low-end IoT devices, which are too resource-constrained to run traditional OS. Thus, the crucial requirements for an IoT OS are to support heterogeneity in hardware architectures, lightweight design, APIs and communication technologies. Furthermore, IoT OS facilitates IoT devices to connect with the Cloud platform and use its services. In fact, IoT OS vendors have also provided Cloud integration capabilities for users, which will drive the integration of Cloud and IoT innovation in the near future. Thanks to the promotion of major corporations and organizations, IoT OS is expected to be quickly applied to IoT applications in the near future. Nevertheless, this also caused some problems. Firstly, Cloud Computing is concerned about the data storage Cloudization, while lacking the effective management and maintenance for software resources under the large-scale heterogeneous IoT devices, especially for the diversified and fragmentary IoT OS resources. Secondly, in the current mode, IoT users can not access a cross-platform and cross-OS service due to the strong customization between IoT OS and their Cloud platform. Thirdly, Cloud Computing has excellent performance in terms of reliability and security on the server side while the performance is not good enough in the IoT node. It is true that the security and reliability of IoT nodes can be effectively improved based on the IoT OS. Meanwhile, as the IoT OS kernel crashes or is attacked, the Cloud server can not fix it well.

**Figure 1:**
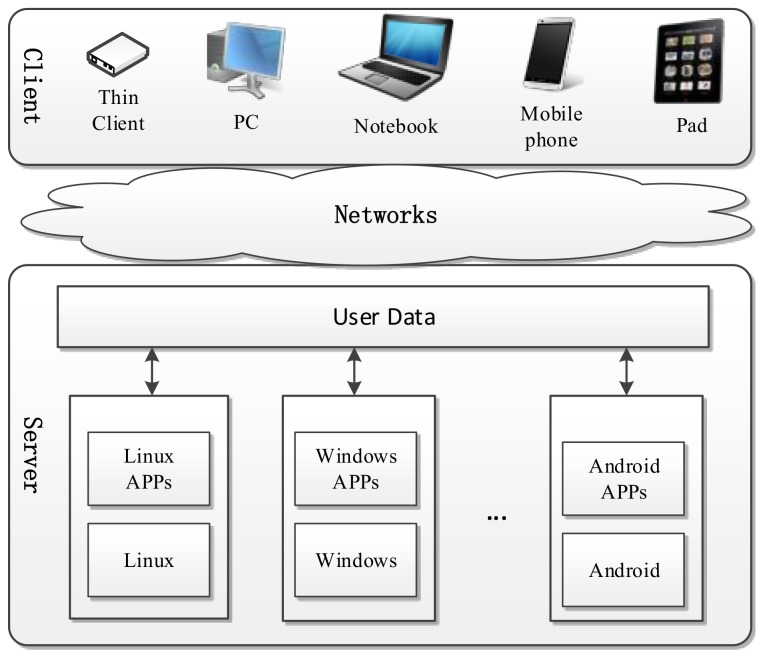
Transparent computing architecture.

Transparent computing was proposed as a new pervasive computing paradigm to provide end-users with transparent services [29]. The kernel ideas of TC include: (1) separation of computation, storage, and management; (2) users are able to freely access the across-platform services on networks across different kinds of heterogeneous terminals; and (3) the service is completely transparent to users, and services are scheduled to the client and performed in a block-streaming way on demand. The detailed illustration of TC architecture is shown in Figure 1.

As shown in Figure 1, a TC based system mainly consists of the transparent server and client. Transparent client is a device with only BIOS (Basic Input Output System) and a small fraction of protocol and management programs, which is not proposed to have any operating system or application software on it. Such a paradigm enables the client terminal to be diverse, secure, light weight, and easy to manage and maintain. Numerous OSs, applications and user data are stored on the transparent server, and loaded to the client terminal via a transparent network in a block streaming way. Therefore, the computation, storage and management are spatio-temporally separated. Users can access the services through cross-terminal and cross-OS operations.

The concept of TC was proposed in 2004, and the authors in [30] provided the concept, architecture and an example of transparent computing. From then on, based on the theory of TC, researchers proposed some representative architecture and developed demonstration applications. The authors in [31] proposed a transparent computing architecture 4VP+, and developed the corresponding client terminal. Users only need to buy a bare computer with a Meta operating system (Meta OS), which is a control platform located on both TC server-side and user-side. It is not only responsible for OS booting, remote accessing, and executing required APPs on the user-side, but also in charge of providing storage and management services for resources (including OSs, APPs and user data), and communication with users. TransOS [32] was proposed on the basis of transparent computing theory, which is defined as a system where clients provide services for users and all traditional operating system codes and applications are centrally stored on network servers. A full-featured, high performance simulation framework for TC named TranSim was proposed in [33]. Using TranSim, the system designer can quickly evaluate the effectiveness of cache schemes along with the system performance. The authors in [34] presented a separating computation and storage strategy. An Intel Corporation (Santa Clara, CA, USA) research team combined a new-generation BIOS named UEFI (Unified Extensible Firmware Interface) with TC architecture [35]. The authors of [36] constructed an optimized algorithm for dependent file fetch middleware in TC platform.

As can be seen from the above surveys, IoT has been combined with some computing paradigms, such as Cloud computing, fog computing and context-aware computing. In addition, a transparent computing system has been implemented on PCs and multiple mobile devices. However, to the best of our knowledge, the explorations and research on integration of IoT and Transparent Computing is at an initial stage. The authors of [37] study an implementation of edge computing, which exploits transparent computing to build scalable IoT platforms. Furthermore, their characteristics are often complementary. IoT can benefit from the TC’s centralized storage and management of resources (OSs, APPs and user data) as well as stream execution on the client side by remotely loading. TC can offer an effective, reliable, secure and cross-OS solution for IoT resource services, and reduce the costs of IoT system deployment and maintenance. On the other hand, TC can benefit from IoT by extending its scope to deal with real world things in a more dynamic and distributed manner. This paper tries to explore a feasible solution for the integration of TC and IoT, and propose a novel industrial IoT architecture based on TC, and present its key technology and evaluation results.

## 3. Architecture for the Resource Service Model of Industrial IoT Based on TC

From the technology perspective, the design of an IoT architecture needs to consider the extensibility, maintainability, modularity, and interoperability among heterogeneous devices. The number of layers of the IoT architecture depends on the application context. In general, the IoT architecture is divided into three layers or four layers. A generic three layers consist of application, transport, and sensing [38]. Michele Nitti et al. [39] proposed a specific architecture for the sustainable tourism application, which foresees a four-level architecture: application, service, virtualization and the real-world layer. The authors of [40] presented an architecture based on IoT and cyber-physical system focused on industrial 4.0, which was divided into four layers: sensors and actuators, communication, entities and services, and the application layer. To realize that transparent computing integrates with industrial IoT, we divide the industrial IoT architecture into four layers: sensing layer, network layer, service and storage layer, and monitoring and management layer. Prior to the presentation of the architecture, our motivation is illustrated through a case study.

### 3.1. A Case Study of Six Systems

The Six Systems is a typical industrial IoT project that is used to ensure the safety of mine production [12]. In the Six Systems, many sensor nodes and collection devices are connected via an industrial Ethernet underground. The sensor node is responsible for receiving signals (like heat, pressure, light or motion, etc.). The sensing data are collected by collection devices and transferred to the ground control center, in order to locate the position of the mine staff and detect hazardous gases.

**Figure 2:**
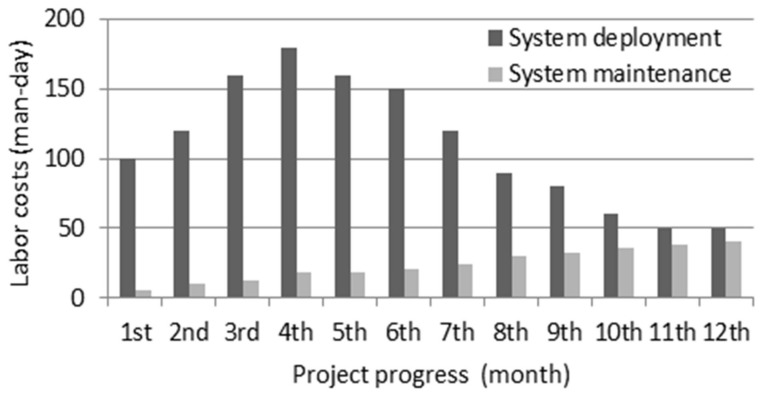
The labor costs of deployment and maintenance in “Six Systems” of Fankou.

Since 2013, our research team has been responsible for the design, deployment and maintenance of Six Systems of Fankou Lead-Zinc Mine Company (located in Shaoguan, China), which is based on the four-layer architecture as mentioned before. We tracked and analyzed the challenge of the industrial IoT system during one year’s work of deployment from 2014 to 2015 and another year’s work of maintenance from 2015 to 2016. Table 1 shows the devices deployed in the underground environment including 102 collection devices and 762 sensor nodes, excluding 2000 RFID identification cards for workers. Based on our experience, the challenges of industrial IoT are summarized as follows:

1. Widespread heterogeneity in devices and resources. Various sensor nodes and collection devices (also called aggregation devices) are used in industrial IoT applications. All of the industrial IoT applications have specific characteristics: heterogeneous and diverse devices and protocols; constrained computing and storage capability; and difficulty in data exchange and sharing. In addition, with the in-depth utilization of IoT, its applications become increasingly rich. All of these elements bring serious challenges to the resource management, interconnectivity and collaboration of IoT devices.
2. The security and reliability of industrial IoT terminal devices is not sufficient for practical application. The current industrial IoT device is almost a bare metal where only a small number of applications are deployed without enough security strategy. On the other hand, a scheme that uses Cloud computing to connect and manage industrial IoT devices and its resources is widely used. However, due to the heterogeneous quality of the device and protocol, the reliability of the Cloud platform connecting to and managing industrial IoT device resources is far from satisfactory.
3. High deployment and maintenance costs for the large-scale sensor nodes and collection devices. IoT application involves many sensor nodes and collection devices. These devices are featured with huge quantities, various types, and scattered locations, which cause high deployment and maintenance costs of an IoT system. Figure 2 shows the labor costs (man-day) of each month in Six Systems deployment and maintenance cycle. As shown in Figure 2, the labor costs of deployment are very high because lots of devices including sensor nodes and collection devices need to be deployed in distributed underground space, and the cost per month is decreasing in the later months during the deployment period. On the other hand, the labor costs of maintenance increase fast after the system gets online. The maintenance costs mainly focused on the collection devices due to their complex functions, high probability of failure and great difficulty of debugging. The sensing device is relatively simple, reliable and cheap. However, the advance of sensing devices, which includes the improvement of computing capabilities, the application of the IoT OS and the increasingly rich functions, also increases the difficulty of management and maintenance.

For the previous two challenges, it is worth mentioning that the birth and development of IoT OS is expected to solve these issues, and our team is concentrating on the application of IoT OS for the industrial IoT. However, the IoT OS also brings new challenges for the IoT system. Firstly, in general, an IoT OS is limited by a vendor that can only connect and access a particular Cloud service. A multiple heterogeneous environment will impact future industrial IoT application development, where information processing, transmission and sharing will become more difficult. Secondly, Cloud computing is concerned with the computing and storage Cloudization, instead of the operating system and software Cloudization. This leads to failing in effective and centralized management for the IoT OS and its application software with Cloud platforms. Finally, the current Cloud platform ensures the IoT users’ reliability and security in this aspect endeavor as using its service, but for the IoT devices’ own reliability and security, the Cloud platform appears to be powerless.

It is true that the problems mentioned above should be tackled in the future industrial IoT system. Unfortunately, most existing works mainly concentrated on the structure of future network, standards of IoT technology and the network protocol, but very few studies have been focused on the challenges mentioned above. Therefore, a new solution that integrates transparent computing into industrial IoT is explored in this paper.

### 3.2. The Architecture

**Figure 3:**
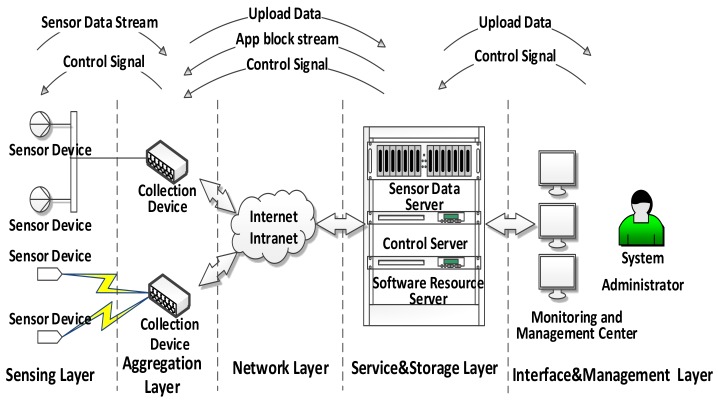
The architecture of industrial IoT based on TC.

This paper proposes a novel system architecture for the industrial IoT application based on transparent computing. Figure 3 shows this architecture consists of sensing layer, aggregation layer, network layer, service and storage layer, and interface and management layer. The network layer is similar to the traditional four-layer IoT application architecture, which is mainly composed of a variety of network communication methods and different industrial fields networking modes. The other four layers of the architecture are described below.

#### 3.2.1. Sensing Layer and Aggregation Layer

The sensing layer is integrated with existing hardware (RFID, intelligent sensors, actuators, etc.) to sense/control the industrial world and acquire data. The aggregation layer, which is responsible for collecting and simply processing the information, and then sends information to the service and storage layer via network layer, is composed of various collection devices and plant floor network module devices. Typically, the sensor is a resource-constrained device that can only run a dedicated IoT OS, and the collection device needs a full-fledged OS such as Linux or BSD (Berkeley Software Distribution). In order to support the separation between software and hardware, and achieve the purpose of OSs and APPs centralized management, the characteristics of devices in the sensing layer are as follows: (1) there is no OS resident on the sensing and aggregation devices, and only a lightweight Pre-OS that is similar to a bootstrap program with a network protocol stack is required to support multiple OS remote booting on demand via network; (2) supporting streaming loading and streaming execution, that is, OSs and APPs are stored on the server side, which are centralized installed, upgraded, assigned and managed; and (3) the data generated by sensing devices are stored in server to ensure the security of the data.

**Figure 4:**
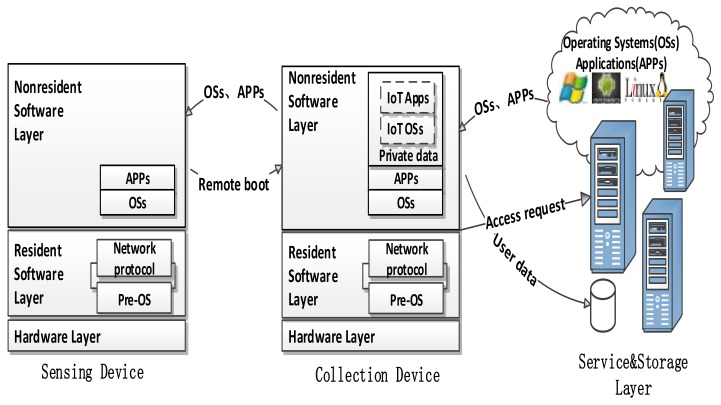
The architecture of the sensing and aggregation layer.

As Figure 4 shows, both the sensing and aggregation device are divided into three sub layers. The hardware layer provides an interface for sensors, collection devices, display and other peripherals; the resident software layer mainly provides network protocol stacks and Pre-OS that can drive hardware and remotely boot various OS kernels; and the nonresident software layer realizes OS loading and program execution in a streaming-block way.

Most tasks of the Pre-OS involve initializing hardware and establishing the appropriate network environment with the network protocol stack. Then, the sensing and collection device select an OS kernel according to the end-user requirements and server configuration, and send requests to the server side for remotely loading the OS kernel and the appropriate file system via the network. Finally, the OS and application files are loaded from the server to nonresident software layer for streaming execution. It should be noted that the IoT OS and APPs of sensing devices are stored and managed in the aggregation device as its private data, and the data generated by sensing devices are first transferred to the aggregation layer with the network protocols supported by the IoT OS, and then stored into the service and storage layer through the data remote storage mechanism.

For these reasons, the requirement of computation and storage capacity of sensing and collection devices is greatly reduced in TC architecture, and the device can be securely and easily managed and maintained. On the other hand, the Pre-OS separates hardware with the operating system, and shields the specific details in the hardware on the bottom layer. Accordingly, multiple different operating systems can be run on the IoT devices.

#### 3.2.2. Service and Storage Layer

**Figure 5:**
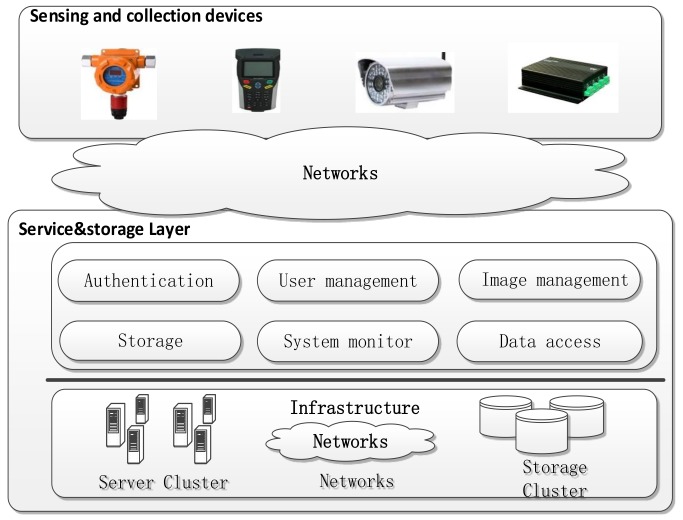
The architecture of the service and storage layer.

On the one hand, the OS images, APPs and private data are stored and managed in the service and storage layer in a centralized way. On the other hand, in this layer, a streamed data access service is required for the multiple OSs and APPs remote loading and execution of the aggregation layer devices. As shown in Figure 5, by means of providing basic services such as authentication, user management, image management and system monitoring, the service and storage layer shields the software resource loading, application configuration and other details for the aggregation layer users, so users only need to pay attention to their own service needs.

1. Resource storage. The resources are classified into three types: OS resource, application resource and personalization data from the aggregation layer devices. All of the resources are stored and uniformly managed in the service and storage layer. Due to the massive and rapid industrial IoT data, an efficient and low redundancy data storage model must be taken into account.
2. Image management. On the service and storage layer, the image as an effective form of resources is easy to be stored and effectively managed. The data access request from the sensing terminal is eventually redirected to the server’s virtual disk images. The image is also the carrier of the end-user data resources, and there is a one-to-one correspondence between the end-user system and virtual disk images. In the face of massive sensing layer devices, how to efficiently create, backup, and upgrade images for sensing layer users is a challenge.
3. Data access. The operating system, applications and personalized data that users need are dynamically obtained from the service and storage layer through the network, while the user’s modifications to these resources can be written in real time to the layer. When numerous sensing devices are connected to the IoT system, the service and storage layer can process the data request quickly and reliably. Because the resources are stored in the server, the data request of aggregation devices is retransmitted to the service and storage layer via networks, then the requests are parsed, and finally it is determined how to handle them.
4. User management and identity authentication. Due to the diversity of industrial IoT devices, users of the device with common attributes often have the same user configuration, and they need to be divided into multiple dimensions. On the other hand, before accessing the TC platform, each user of the aggregation layer must be authenticated, and then enjoy the cross-terminal and seamless personalized service.

When the Pre-OS in the device of aggregation layer is started, it will encapsulate the instruction that boots the operating system needed, then transmits it to the service and storage layer via networks. The service and storage layer responds to and processes the request, and loads the corresponding image resources to the aggregation layer device for execution in a block-stream way. After the operating system is started, the resource service request sent by the aggregation layer device is redirected to the service and storage layer by the Vdisk driver, so that the device can access the corresponding instructions and data, which are subsequently scheduled to the device and performed with the remote resource access protocol.

#### 3.2.3. Interface & Management Layer

The interface and management layer is used to describe the specifications between applications and services. There is also a necessity for an interface and management layer to simplify the management and interconnection of things. It can be seen as a set of services that support interaction with the entire system. This layer can use the data of service and storage layer for analyzing and displaying momentarily after a certain user privilege certification. As a result, the system can analyze and monitor the indexes of industrial environment all the time, and implement real-time monitoring of the working status of sensing layer devices. The update and maintenance of system software resources mainly depend on the resource scheduling service of the service and storage layer. The system administrators only need to assign the tasks, and then wait and confirm the returning execution results. The specific operation of software resources is processed by the resource scheduling service.

## 4. Key Technologies of the Resource Service Model

The resource service model based on TC focuses on providing the unified resources storage, management and access service for various heterogeneous industrial IoT devices. Generally, all of the resources in the service and storage layer are organized and stored in the form of virtual disk. Then, the collection and sensing devices utilize appropriate methods to access these remote resources. Therefore, the key technologies include virtual disk storage model, structure and data access method.

### 4.1. The Virtual Disk Storage Model in the Service & Storage Layer

The ubiquitous sensors, collection, and other devices involved in the industrial IoT systems can generate data rapidly so that the volume of the data is very large and can increase rapidly. In addition, various operating systems of sensors and collection devices need to be stored and managed at the service and storage layer. Thus, a data storage model with the ability of efficiently storing and managing massive industrial IoT data is required in the layer. In the TC pattern, all the resources are organized and stored on the service and storage layer in the form of virtual disk (Vdisk) data. Thus, a multi-layer and chained model for Vdisk storage is achieved. Based on the level of sharing degree, data resources in Vdisk are divided into three categories:

1. System shared resources. This type contains the data that belong to or are related to the operating system, including various supporting software and managing tools, which owns the highest sharing degree and is available for all users. The image constructed with this type of resource is called system virtual disk image (S_VDI).
2. Application-group resources. All the data related to the application software is categorized as this type. Applications with similar properties would be classified as the same group. Those data are shared by users of the same group and the corresponding image is called a group virtual disk image (G_VDI).
3. User-customized resources. This mainly means that the data belong to a certain user, which normally includes user private files and preference information of application. The data sharing degree is the lowest and are only accessible for the owner. The corresponding image is called a user virtual disk image (U_VDI).

**Figure 6:**
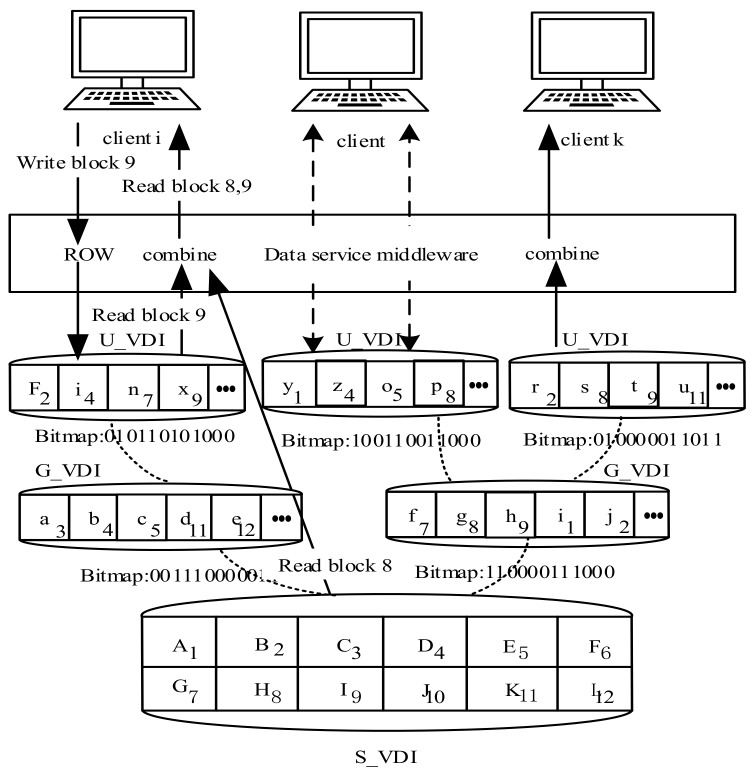
The Vdisk storage model and accessing mechanism.

As Figure 6 shows, the top level of the tree is the system virtual disk. It is the highest sharing level, where the data are available for each terminal user. The group virtual disk is the second level of the tree, where the data are shared in the same user group. The bottom of the tree is the user virtual disk, where little allocated storage space is required for upper level data editing, and the remaining space is for users’ private data.

In such a Vdisk data storage model, the entire data storage and access mechanisms are transparent to the user. From the user’s point of view, they own the "local disk" in an exclusive way, which contains the operating system, applications and private data. Actually, the "local disk" is divided into multiple parts on the server side. S_VDI and G_VDI are multi-user sharing images, while only U_VDI stores the exclusive private data for users.

As the core component of the service and storage layer, the data service middleware is mainly responsible for receiving and processing I/O requests from the collection device, and calling the underlying distributed virtual file system interface to help end-users achieve the actual data access. It is composed of the receiving and classifying requests, authenticating, scheduling, hierarchical caching, coding/decoding, and data response modules. Among them, the receiving and classifying requests module is in charge of receiving data requests from the terminal and dividing them into different scheduling queues; the authenticating module is mainly used to determine the legitimacy of the access connection and request method that is to be sent to the server; the scheduling module needs to ensure the reliability of the service when many terminals are connected to the platform at the same time; the primary responsibility of the hierarchical caching module is to place the data blocks that are accessed frequently in the server cache, thus speeding up data access; the coding/decoding module takes charge of unifying the data format, while ensuring the security of data transmission; and the data response module is responsible for responding to all data requests from terminals.

### 4.2. Virtual Disk Structure for the Service & Storage Layer

Although the Vdisk storage model in transparent computing could solve the problem of consistency of multi-user Vdisk shared data, a certain suitable Vdisk storage structure is necessary to support the model to achieve high efficiency of accessing and sharing in this computing system. This paper presents a bitmap-based [41] Vdisk structure technique to reach relatively high accessing efficiency with low granularity for users.

This structure consists of Header, Bitmap, Q_table and data. The Header, Bitmap and Q_table construct the metadata file, while the Data part constructs the data file. The metadata and data file are able to store continuously or separately. In order to achieve faster location, based on the different granularity, two types of sequences could be obtained.

**Definition** **1.** Block Sequence (BS): BS={B1,B2,…,Bn}, it is constructed by continuous blocks with the same size. The size of each block is B_unit. BS is the granularity of sharing and editing on the virtual disk.

**Definition** **2.** Chunk Sequence (CS): CS={C1,C2,…,Cn}, similar to BS, the CS consists of continuous chunk with the same size and each chunk is a BS with the fixed length, $C_{i}=\left\{B1,B2,\ldots ,Bn\right\}$. Dividing granularity of CS is C_unit, which is the basic unit of data searching.

**Figure 7:**
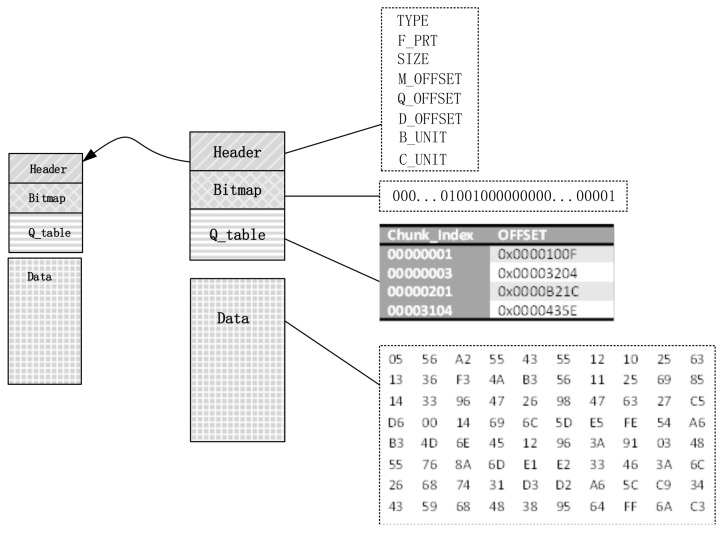
The Vdisk storage structure.

The virtual disk storage structure is shown as Figure 7. The header is located at the start of the virtual disk, which records the fundamental information of the whole virtual disk. Its member variables and description are shown in Table 2.

The Bitmap is used to record the location of edited blocks. When initializing the image node, a space with size of Bitmap_size that can be calculated by Equation (1) would be allocated and cleared as 0. Every bit in bitmap is corresponding to every block in the *BS*. After initialization, the corresponding bit would be set to 1 if the father node block sequence is rewritten:

(1) Bitmap_size=SIZE/B_unit.

Query table (Q_table) makes the index of the chunk map to the offset of the chunk in Data part, so that the query speed is enhanced. The size of the table is changeable and adjusted dynamically according to the quantity of rewritten data. Only rewritten chunks are recorded in the table. The data part is utilized to store the edited data that are in its father node. Then, the edited with shared data can be distinguished with those in the father node. The sequence of storage in the Data region of a certain node should be the same with the sequence in the terminal virtual disk view.

### 4.3. The Data Access Method in the Sensing Layer and Aggregation Layer

In our TC-based industrial IoT system model, there are two layers. One is the aggregation layer, and the other is the sensing layer. From the perspective of an aggregation layer user, there is no difference between Vdisks and local hard disks. However, Vdisk is just a virtualized logical disk device in the collection terminal, and its actual data reside at the service and storage layer. Data in Vdisk are fetched to the local for execution on demand. First, the aggregation layer devices need to establish a network environment to connect with the transparent server in the service and storage layer. Second, since there is no local operating system when the aggregation layer device is started, in order to remotely start an OS from the TC server, and read the OS instructions from the Vdisk images, a set of protocols are needed, termed as MRBP (Multi-OS Remote Booting Protocol). It extends the traditional BIOS function by integrating a network driver. Finally, when the client OS runs after starting, the OS can access the data (read or write) from the Vdisk as from a normal hard disk with the NSAP (Network Service Access Protocol) [42].

Here, an example is given to illustrate the data access process of the aggregation layer user from Vdisk images. In Figure 6, when user *i* tries to write data block 9, the request will be redirected to U_VDI that is corresponding to the user by the data service middleware, and the bitmap index marks the corresponding bit that has been changed. After this editing, once user *i* attempted to change data in block 9, original data in block would be replaced directly and it is not necessary to change the bitmap. Furthermore, when terminal user *i* requests data block 8 and 9 because the edited block 9 is stored in U_VDI, the request will be redirected to corresponding U_VDI. Data block 8 is not marked in the bitmaps of U_VDI and G_VDI, so the request is directed to S_VDI. Finally, all the required data blocks would be recombined and returned to users.

To give more details, we give the Algorithm 1 to illustrate the process that read access to Vdisk image from the aggregation layer. First of all, it is necessary to calculate the Block_index (the block number), Chunk_index (the chunk number) and Start_no (the start number of the block in the owning chunk) of the request data block according to the offset and length. They can be calculated by the following equations:

(2) Block_index=⌊offset/B_unit⌋,

(3) Chunk_index=⌊offset/C_unit⌋,

(4) $$Start\_no=\lfloor \frac{offset\%C\_unit}{B\_unit}\rfloor .$$

Secondly, querying the bitmap of the user node to determine whether the block to be read is rewritten by the user. If it has been rewritten, try to achieve this access through the caching mechanism. If the block has not been rewritten, query the bitmap of its parent node. If the block to be read is rewritten in the parent node, read the block from the data area. Otherwise, read the block from the data area of top-level node. Finally, if the data block requested this time has been read completely, then merge the content that has been read.

**Algorithm 1:** Algorithm for reading blocks of Vdisk at aggregation layer. **Input** :     Vdisk Id: vid;     Block offset to read: off;     Block length to read: len; **Output**:     The data to be read: D{0…n−1};1 initialize D{0…n−1};2 **for**
*i=0 to len*
**do**3  Calculate the Block_index and Chunk_index of $Block_{vid}^{offset+i}$ according to the offset and length;4  Calculate the Start_no of $Block_{vid}^{offset+i}$ in the associated chunk;5  Query the bitmap of the user;    **if**
*the block to be read is rewritten*
**then**    Look up for the block in local cache;    **if**
*find in the cache*
**then**     $D\left[i\right]=Block_{vid}^{offset+i}$;    **else**     Fetch Block from the remote server;     Update the local cache;    **else**     **for**
*each parent node of the current access node*
**do**     Query the bitmap of the parent node;     **if**
*the block to be read is rewritten in the parent node*
**then**      Read the block from the Data area;      break;     **end**    **end**    **if**
*the data has all been read*
**then**    Merge the content of read;    **else**    goto the step 3;6 **end**

On the other hand, for the sensing layer devices data access, the IoT OS and its APPs that they need are stored as the private data at the aggregation layer. Therefore, an MRBP protocol is needed to be integrated into the bootstrap of the IoT OS. However, unlike the aggregation layer devices, the sensing layer devices are responsible for sensing and gathering information, and then transmitting them to the aggregation layer through the IoT network protocol. Fortunately, the information can be transferred to the aggregation layer with the lightweight network protocol that IoT supports, and then stored into the service and storage layer through the Vdisk remote storage mechanism of the aggregation layer.

According to the above idea, we also proposed a general solution for the remote booting and management of the lightweight IoT OS correspondingly. The IoT OS images are distributed on remote servers, while the bootloader of the IoT terminal device specifies the initial URI (Uniform Resource Identifier) with the corresponding OSs image loading information. After the network configuration is initialized, the bootloader obtains the corresponding remote server IP address according to the initial URI domain name resolution, establishes a TCP connection with the remote server, and then sends a network service request (such as HTTP, HTTPS, FTTP, etc.) to load the OS image. If the desired image does not exist on the server, the service request is redirected to the nearest server node, and so on, until the image is found. Finally, the initial URI in the boot program is replaced by the URI specified by the latest redirected message.

## 5. Implementation and Experimental Evaluation

We constructed a prototype system that implements the TC-based resource service model for industrial IoT. For simplicity, it is named TCIIoT. We first give the qualitative evaluation for the proposed resource service model. Compared with the general Cloud-based IoT platform, our model has some advantages in the following aspects. Firstly, the model not only focuses on data Cloudization, but also emphasizes the Cloudization of software resources, especially the operating system resources. Hence, it improves the manageability of the software resources for the various IoT devices. Secondly, benefiting from the decoupling between physical device and OSs, heterogeneous IoT devices can support the execution of a variety of OSs, thus the scalability of IoT devices are enhanced. Thirdly, due to the separation of storage and computing, there is no need to install any OSs and APPs on IoT devices, so a large number of system deployment and maintenance costs are reduced. Finally, owing to the stream execution of the program on the IoT device, it is difficult for the malicious program to reside on the device, even if the system collapses, it can be quickly restored. As a result, the reliability and security of the IoT device are greatly improved. Next, we will introduce the details of implementation procedure hierarchically, and evaluate the system performance and compare it to the others simply with several experiments. We also analyze and discuss the evaluation results and their impact on the future industrial IoT in detail.

### 5.1. IoT OS Remote Booting in the Sensing Layer

In this section, we focus on the remotely booting IoT OS that is designed for low-power and resource-constrained IoT devices and applications from the aggregation layer for streaming execution. The IoT OS we adopted is Zephyr [28], which is an open source IoT real-time operation system developed by the Linux Foundation. The Zephyr kernel only requires at least 8 KB memory and provides micro-kernels, even nano-kernel mechanisms. More importantly, Zephyr provides connectivity protocols optimized for low powered, small memory footprint devices, where Bluetooth and WiFi as well as 802.15.4 has been supported. In the future, more standards like 6Lowpan, CoAP, IPv6 and NFC (Near Field Communication) will be supported.

The hardware platform applied here is an x86 mobile device, Minnow Board Max integrating an Intel Atom E3800 processor, which is used to simulate an intelligent terminal node of the industrial IoT. The network bandwidth of the experimental environment is 100 Mbps. The size of the Zephyr microkernel after the compilation is 18 KB. Zephyr can be locally booted by grub2, but grub2 can not be run on such a mobile platform and support remote booting. Therefore, we first build a HTTP server in the OS of aggregation layer devices, where the Zephyr kernel and APPs are stored. Then, a new entry is set up in the grub2, where essential remote booting configurations and method code are written. Finally, we compile the new grub2 to UEFI boot file. We tested the performance of Zephyr remote boot performance with our method, including the booting time and the consumption of bandwidth. Each test was repeated five times, and these averages were finally obtained. The results are shown in Table 3. The results indicate that the remote booting time of the Zephyr is similar to the local launch, and the bandwidth consumption is also at a lower level, and approaches linear growth with the increase in the number of devices. Furthermore, such a solution that can remotely boot and load the IoT OS using a transparent computing idea makes the manageability, security and reliability of IoT terminal devices effectively improved.

### 5.2. The Implementation and Performance of the Aggregation Layer

The aggregation layer supports both Windows 7 and CentOS 6.5. In our implementation, the MRBP is based on the Intel gPXE protocol for sending remote booting requests. We specifically designed and developed the wireless network driver for the mobile Internet application environment, making the mobile collection devices also remotely load resources. The implementation of NSAP is based on the iSCSI (Internet Small Computer System Interface) protocol. Two different Vdisk drivers for the Windows and Linux are implemented as an iSCSI port device driver at the block level, respectively.

In order to test the performance of the aggregation layer, we try to load the CentOS and Windows 7 in succession to the collection devices, and take the user experience (latency) as a measure. Without loss of generality, we use PC and PAD as an aggregation layer device in this experiment. The PC is an Intel Pentium 2.93 GHz machine, each with 2 GB DDR2 and a 1000 Mbps onboard network card. The PAD is an Intel Bay Trail SoC (System on Chip) machine, each with 2 GB DDR2 and 1000 Mbps onboard network card. Their hardware configuration is very similar to the collection device in industrial IoT.

**Figure 8:**
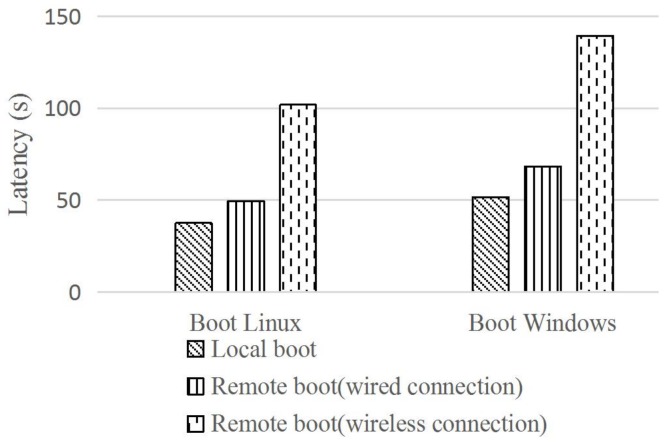
Multi-OS remote booting on-demand in aggregation layer.

Figure 8 shows that we are able to load and boot Linux (9.8 GB) and Windows (15.5 GB) in the aggregation layer. Furthermore, we compared the OS booting time of local boot and remote boot under both the wired and wireless networks. Under the wired connection is close to that of local boot with the time of 49.6 s (Linux) and 68.4 s (Windows). While being under wireless conditions, the latency is more than two times the wired connection with the time of 101.9 s (Linux) and 139.5 s (Windows), yet still acceptable. However, it is worth mentioning that all of these tests are in the absence of any cache, and the results are the average of five trials. We also tested the performance with the same devices that added a cache mechanism. The results show that both the Linux and Windows of booting time are between 70 and 80 s, which is a user experience similar to an interaction with a local machine. Actually, most of the collection devices in industrial IoT have some cache functions.

Table 4 illustrates the performance of executing some applications or operations in Linux and Windows, respectively. We compared the latency of a local device and collection device in our proposed model under the wired environment in the following three aspects: file coping, file downloading, and application launching. The file copying is carried out among different logical disks under the same user view. Files can be downloaded from a FTP server in the LAN. As for the application launching, it is processed by loading applications from the server to the terminal device, so that users can execute the applications on the terminals. The results show that the response time of the two devices as executing the operation or application that required data is relatively small. However, if the amount of data that the operation or application required is larger, then the response time of the collection device is not that ideal, but it is still acceptable. This is because the response time mainly depends on the network condition in such a scenario. The network conditions are not a bottleneck for data transmission in the case of a small amount of data requests, such as copying a 10 MB file and launching a Web browser. Consequently, the performance is determined by the disk throughput. In general, the throughput of the collection device is greater than that of the local device, so the performance on the collection device is also better.

### 5.3. The Performance Analysis for the Service & the Storage Layer

The primary function of the service and storage layer is to store and manage virtual disk data for each user. The virtual disk storage model is implemented in the Java language and deployed in the Linux of the server. The reading and writing performance of the virtual disk is an important index to measure the performance of the service and storage layer.

In this test, through the collection device to read and write the virtual disk file data with different request sizes, get their throughput, and compared with the local disk under the same experimental conditions. The server machine is configured with an Intel Xeon(R) 2.5 GHz CPU, 64 GB Dual DDR2, 15,000 rpm SATA hard disk, and 1 GB onboard network card. The collection device is Lenovo E49AL (Beijing, China) with 4800 rpm hard disk and CentOS. There is no use of PAD to do the test because we found that under the environment of wireless network PAD network is not stable, it will become the bottleneck of the virtual disk read and write speed, thus affecting the real virtual disk read and write speed results.

**Figure 9:**
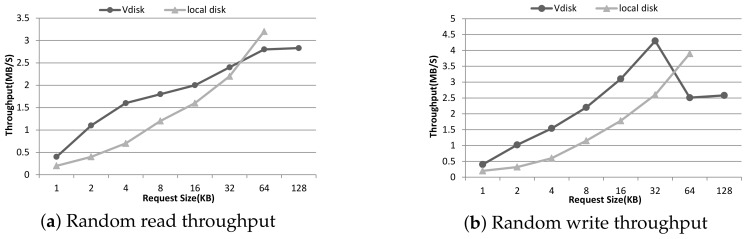
The performance results on random read throughput (**a**) and write throughput (**b**) of the service and storage layer.

CrystalDiskMark tool is used in the test, and test data size is 500 M. The results shown in Figure 9 are the average of five trials. Because the standard deviations are small (less than 5 %), they are not plotted here. We also compared the throughput with that of a regular PC’s local hard disk. As seen from Figure 9a, for read access, the Vdisk throughput in our model increases with request size and is higher than the local disk, but decreasing when the request size is large than 64 KB, which is the maximum size delivered by one Vdisk driver. In addition, when the request size is larger, the network communication dominates the latency, for that a large request size will cause several servicerequests. Meanwhile, the cache module in our system is disabled in this experiment, but the caching of the operating system on the server side can not be disabled. Because the operating system cache is a memory cache, and its size is small, it plays a more important role when there is a small amount of request data. As a result, the Vdisk performance is higher than the local disk when the request size is small. The write access shown in Figure 9b is similar to that of the read access, but decreasing at the size of 32 KB, which is also the maximum size that can be delivered. It should be noted that the random write throughput was better than random read throughput when the request size was greater than 8 KB, and this may benefit from the write-behind mechanism that can return the request response immediately without carrying the real disk operations.

On the other hand, the disk usage on TCIIoT and the local disk were evaluated and analyzed, respectively. In this experiment, we used the CentOS image with the size of 9.8 G, which includes office and entertainment groups, and added five users to each group. With the increase of terminals, the results of disk usage are shown in Table 5. The occupancy space on the local disk almost linearly grows as the number of terminals increases, while the disk space of TCIIoT increases very slowly due to the sharing of operating systems and applications of groups by multiple terminals. Meanwhile, we used five mobile terminals and PC to test the data reading/writing performance of the service and storage layer. By reading and writing files to the service and storage layer with different sizes, we tested the speed of data reading and writing under different terminal types with the CentOS’s own disk tool dd, and compared it with the data access method using sockets. The average of the five experiments is shown in Table 6. Overall, the performance of the access method with sockets is a little better than that of ITCIIoT, but the difference is very small. The performance of the PC terminal is better than the mobile terminal because of better network conditions. In addition, in all cases, the speed of writing is higher than that of reading due to the write-behind mechanism. However, as the amount of written data increases, the writing speed decreases obviously. The reason is that there are not enough caching spaces and the writing request can not be responded in time.

## 6. Conclusions

As exploratory research, this paper introduced a new system model for the future industrial IoT application based on transparent computing. In this model, sensing devices had no need to store novel IoT OS, and they can boot multiple OSs and applications on demand by the decoupling hardware and OSs. The aggregation layer provided centralized management for OSs, applications, and the sensing data, and transferred them to the service and storage layer for storing, which gave a new view to addressing the challenges faced by the IoT development.

Aimed to support numerous devices under the new system model, the model took a centralized resource management method to manage and maintain all resources of the collection devices; in addition, a distributed architecture was used to store these resources. As the number of devices increases, the system could increase the number of servers in a linear manner to support the expected scale of IoT applications. As from the experimental results, the network bandwidth is the major factor limiting the number of devices mounted to one server. This paper discussed the maintenance cost of collection devices with regard to the number devices mounted on the server. We also compared the cost to the average expense of each single collection device in the case of the Fankou Six system. We demonstrated the advantage of the new system model of the industrial IoT in saving the expenses as well as concentrating the resources. However, when integrating the transparent computing into industrial IoT, we are still facing several new challenges caused by the network environment and resource-constrained terminal devices. Firstly, frequent data requests and transmissions between the client and server make TC very sensitive to the network environment, while the IoT wireless communication is unstable, speed-limited and costly. Thus, it could have significantly adverse impacts in terms of guaranteeing the user experience. Furthermore, TC suffers from inadequate capacity of data analysis and processing. Finally, because the computation task is performed on the IoT terminal device, it is likely to be more energy-consuming. To this end, future work includes building a suitable caching mechanism for our system model, try to apply big data analysis and processing technology of Cloud computing into TC for industrial IoT and partition the computation tasks on the IoT devices and edge servers to save the energy of IoT devices.

## Figures and Tables

**Table 1:** Devices used in the Fankou Six System.

Device Type	Device Name	Number
Aggregation layer device	Collection substation in personnel regional positioning system	52
	Collection substation in monitoring and supervision system	50
Sensor layer device	Card reader	301
	Wind speed sensor	138
	Wind pressure sensor	6
	H2S sensor	24
	SO2 sensor	24
	On-off sensor	69

**Table 2:** The member variables of the header.

Member	Description
TYPE	64-bit integer to uniquely identify a certain virtual disk node to distinguish with others
F_PTR	A pointer to the father node image, when pointing to the father node, this node could share data in the father image and only stores the editing part of the father node image. This property of system type node is null.
SIZE	It indicates size of image node, which is equal to the total size that the bitmap could cover. The unit is bytes.
M_OFFSET	It represents the offset of Bitmap region from the start position, the Bitmap follows the Head, while the size of Head is fixed; hence, this value should also be fixed.
Q_OFFSET	It records the offset of Q_table part from image start, which could help to locate the Q_table faster.
D_OFFSET	It indicates the offset of Data part from the image start.
B_UNIT	The basic unit of dividing Block in *BS*.
C_UNIT	The basic unit of dividing Chunk in *CS*, C_unit=n×B_unit .

**Table 3:** Zephyr remote booting performance.

	Booting Time	Peak Bandwidth	Average Bandwidth
One client	1.26 s	717 KB/s	364 KB/s
Three clients	1.30 s	1432 KB/s	748 KB/s
Five clients	1.34 s	2043 KB/s	1345 KB/s

**Table 4:** Operation latency in Linux and Windows (s).

Operation	Local Device		Collection Device	
	Linux	Windows	Linux	Windows
Copy a file				
10 M	1.21	1.76	0.94	1.21
50 M	2.33	2.95	2.79	3.97
Download a file				
10 M	9.34	10.03	10.22	11.20
50 M	53.17	58.36	62.36	68.89
Launch an APP				
Web browser	1.89	1.93	1.65	1.71
Disk detection tool	2.78	3.14	2.97	4.57

**Table 5:** The disk usage comparison with the increasing of terminals (GB).

Storage Method	One Terminal	Two Terminals	Three Terminals	Five Terminals	Ten Terminals
TCIIoT	12.5	14.1	16.2	19.8	25.3
Local disk	9.8	22.6	33.4	57.2	116.3

**Table 6:** The speed of reading and writing results (MB/S).

Request Method	Terminal Type	50 M		500 M		1000 M	
		Read	Write	Read	Write	Read	Write
TCIIoT	Mobile terminal	7.2	13.0	7.0	12.2	5.8	8.2
	PC terminal	10.3	20.1	13.0	18.4	12.3	13.6
Socket	Mobile terminal	7.6	13.8	7.8	12.9	6.3	9.4
	PC terminal	11.5	21.4	13.6	19.2	13.1	14.3
